# The Response of Human Skin Commensal Bacteria as a Reflection of UV Radiation: UV-B Decreases Porphyrin Production

**DOI:** 10.1371/journal.pone.0047798

**Published:** 2012-10-25

**Authors:** Yanhan Wang, Wenhong Zhu, Muya Shu, Yong Jiang, Richard L. Gallo, Yu-Tsueng Liu, Chun-Ming Huang

**Affiliations:** 1 Division of Dermatology, Department of Medicine, University of California San Diego, San Diego, California, United States of America; 2 Sanford-Burnham Institute for Medical Research, La Jolla, California, United States of America; 3 Surface Bioadvances Inc., San Diego, California, United States of America; 4 Moores Cancer Center, University of California San Diego, La Jolla, California, United States of America; MGH, MMS, United States of America

## Abstract

Recent global radiation fears reflect the urgent need for a new modality that can simply determine if people are in a radiation risk of developing cancer and other illnesses. Ultraviolet (UV) radiation has been thought to be the major risk factor for most skin cancers. Although various biomarkers derived from the responses of human cells have been revealed, detection of these biomarkers is cumbersome, probably requires taking live human tissues, and varies significantly depending on human immune status. Here we hypothesize that the reaction of *Propionibacterium acnes* (*P. acnes*), a human resident skin commensal, to UV radiation can serve as early surrogate markers for radiation risk because the bacteria are immediately responsive to radiation. In addition, the bacteria can be readily accessible and exposed to the same field of radiation as human body. To test our hypothesis, *P. acnes* was exposed to UV-B radiation. The production of porphyrins in *P. acnes* was significantly reduced with increasing doses of UV-B. The porphyrin reduction can be detected in both *P. acnes* and human skin bacterial isolates. Exposure of UV-B to *P. acnes*- inoculated mice led to a significant decrease in porphyrin production in a single colony of *P. acnes* and simultaneously induced the formation of cyclobutane pyrimidine dimers (CPD) in the epidermal layers of mouse skin. Mass spectrometric analysis via a linear trap quadrupole (LTQ)-Orbitrap XL showed that five peptides including an internal peptide (THLPTGIVVSCQNER) of a peptide chain release factor 2 (RF2) were oxidized by UV-B. Seven peptides including three internal peptides of 60 kDa chaperonin 1 were de-oxidized by UV-B. When compared to UV-B, gamma radiation also decreased the porphyrin production of *P. acnes* in a dose-dependent manner, but induced a different signature of protein oxidation/de-oxidation. We highlight that uncovering response of skin microbiome to radiation will facilitate the development of pre-symptomatic diagnosis of radiation risk in a battlefield exposure, nuclear accidents, terrorist attacks, or cancer imaging/therapy.

## Introduction

There is a need to develop a simple biodosimetry that potentially can predict the risk of radiation. Although many radiation detectors are available, it is impossible for people to carry these detectors in all the time of their lives since radiation accidents and risks are unpredictable. There are two distinct types of radiation; ionizing and non-ionizing. Ultraviolet (UV), a non-ionizing radiation from sunlight is thought to be the major risk for most skin cancers [1]. UV radiation is considered the main cause of non-melanoma skin cancers (NMSC), including basal cell carcinoma (BCC) and squamous cell carcinoma (SCC). These cancers strike more than a million and more than 250,000 Americans, respectively, each year. Traditionally, a biological marker of exposure, or biomarker, is defined as “cellular, biochemical, or molecular alterations that are measurable in biological media such as human tissues cells or fluids” [2]. Following this traditional definition, scientists have identified many UV-mediated biological markers. These markers include DNA damage responses (e.g. cyclobutane pyrimidine dimers), the induction of transcription factors (e.g. AP-1, NF-kB, and p53) [3], [4] and regulation of cytokines [e.g. tumour necrosis factor (TNF)-alpha] in skin cells [5]. However, detection of these markers is not only cumbersome but also time consuming. It is also required to take tissues from peoples by skilled personnel. Most importantly, biomarkers identified from tissues/organs may be not radiation-specific since they can change in response to other physiological conditions such as illness and aging. Furthermore, people in healthy conditions are normally unwilling to provide their skin samples to clinicians for determination of whether they are in risk of developing skin cancers. Thus, the feasibility of using biomarkers identified from skin cells as predictors for cancer initiation in clinical practice may be limited.

Skin commensal bacteria mostly reside on the surface of keratinocytes of the human epidermis. These commensal bacteria receive the same UV radiation exposure as skin keratinocytes. Therefore, a positive correlation may exist between skin commensals and human tissues for dose-dependent genotoxic responses. Here, we hypothesize that the detection of the risk of UV radiation can be achieved by monitoring the response of skin commensal bacteria. Detection of UV radiation exposure using skin commensals is simple since sample collection from the surface of the skin is readily accessible and required minimally trained personnel. *Propionibacterium acnes* (*P. acnes*), predominated (>60% of total bacteria) in the facial skins [6], [7] was selected to test our hypothesis. Nearly everyone hosts *P. acnes* [7], [8] which accounts for approximately half of the total skin microbiome [9], with an estimated density of 10^2^ to 10^5–6^ cm^2^ [10]. In addition, the commensal bacterium was predomaintly found on the foreheads [10^3^ to 10^7^ colony-forming unit (CFU)] of normal individuals in Seattle and Alaska either winter or summer [11], [12] suggesting no differences of bacterial colonization between different populations during various seasons. There are approximately ten times as many microbes as human cells in a human body [6]. The skin is the human body's largest organ, colonized by a diverse milieu of microbes, most of which are commensals since they are harmless or even beneficial to their host [13], [14], [15]. These skin commensal bacteria are obviously the most exposed living organisms in human body to sun radiations. Analysis of the response of human skin commensal bacteria to sun radiation has the added advantage of giving a response profile over time because bacteria can be collected and pooled over set periods, as opposed to the snapshot obtained from a single tissue (e.g. skin and blood) sample. Thus, detection of the change in skin microbiome may uncover valuable biomarkers or biodosimetry for UV-induced skin cancers.

## Results

### Porphyrins are Detectable in *P. acnes* and Human Skins

**Figure 1:**
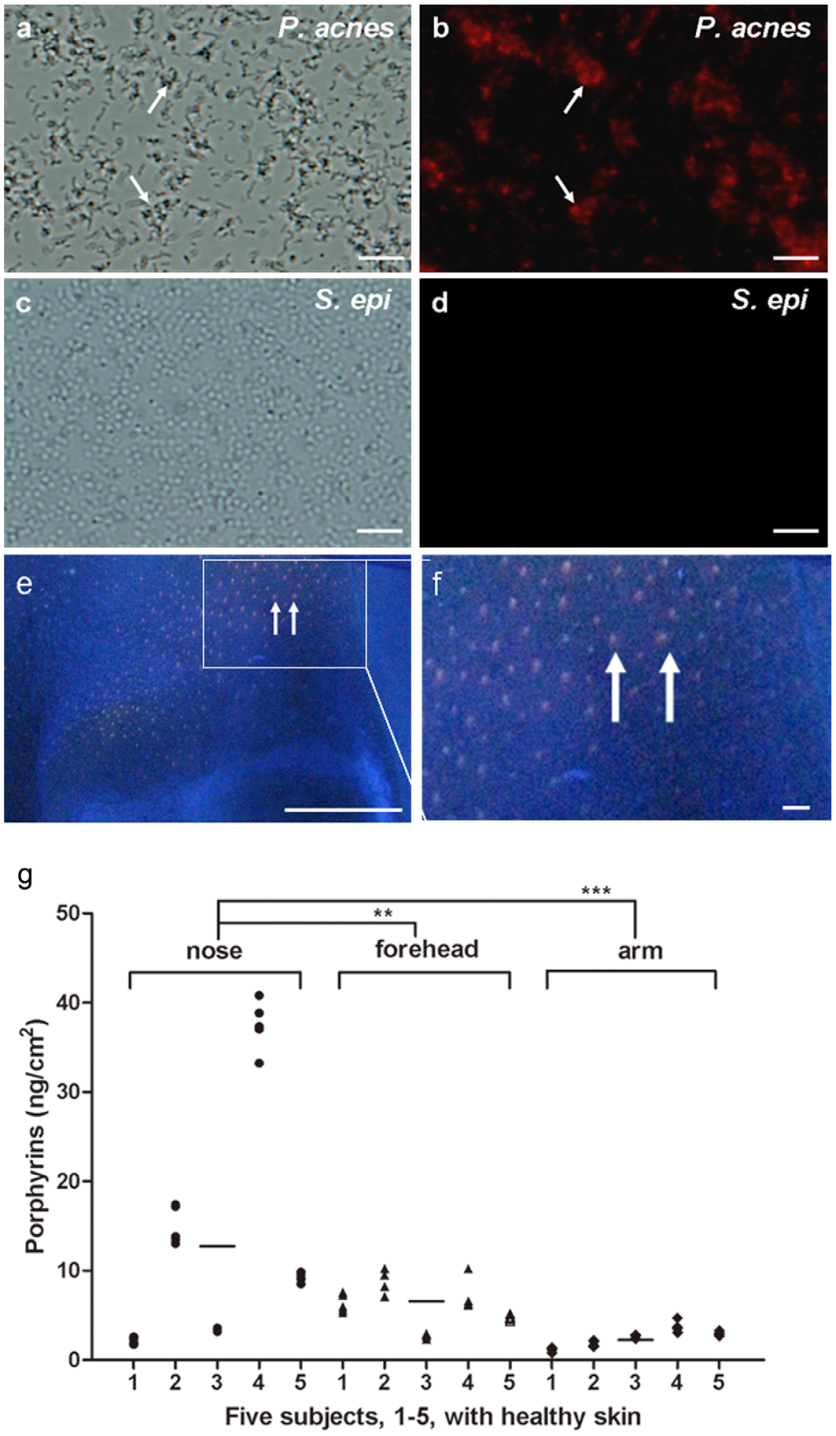
Detection of porphyrins in *P. acnes* and human skins. To visualize porphyrin-producing bacteria (arrows), *P. acnes* (a, b) or *S. epidermidis* (c, d) (2×10^8^ CFU) was incubated with ALA for 4 h and observed under microscopes. Bright-field (a, c)**/**fluorescent (b, d) overlay of bacteria were presented. Bar = 2 µm. A Wood's lamp with an UV light source was used to image the auto-fluorescent *P. acnes* (red fluorescence, arrows) on the surface of nose (e). An area (square) of nose was amplified (f). Bar = 1 cm. The amounts (ng/cm^2^) of prophyrins on the surfaces of noses, foreheads, and arms of five volunteers (1–5) with healthy skins were compared (g). **P<0.01 or ***P<0.001 was evaluated using two-tailed *t*-tests. Data are the mean ± SD of three separate experiments.

*P. acnes* can produce porphyrins, mostly coproporphyrin III (CpIII) and uroporphyrin III (UpIII) [16], [17], [18]. The endogenous porphyrins in *P. acnes* absorb light in the near UV (150–400 nm) and the visible (580–650 nm) regions [19], [20]. It has been reported that irradiation of *P. acnes* with blue light (400–420 nm) leads to photo-excitation of bacterial porphyrins, singlet oxygen production and eventually bacterial destruction [21]. A limiting factor in the porphyrin biosynthesis in several prokaryotic cells is the formation of 5-aminolevulinic acid (ALA). Exogenous addition of ALA into bacteria results in an increase in the concentration of endogenous CpIII and UpIII after bacteria take up the ALA [22]. To measure porphyrins in skin bacteria, ALA (1 mM) was added into the culture of *P. acnes* [American Type Culture Collection (ATCC)6919] or *Staphylococcus epidermidis* (*S. epidermidis*) (ATCC12228), two most predominant bacteria in human skin. As shown in Figure 1 a–d, fluorescence derived from porphyrins (CpIII and UpIII) is detectable in *P. acnes*, but not *S. epidermidis*. A Wood's lamp emitting long wave UV has been used to visualize *P. acnes* in human faces. Red fluorescence of porphyrins in *P. acnes* in human facial skin was more predominant on the surface of nose (Figure 1 e, f) than forehead (data not shown). Consistently, fluorescence spectrometric analysis demonstrated that the amounts of porphyrins around the surface of nose are significantly greater than those on the surfaces of forehead and arm (Figure 1 g). The results are in agreement with previous findings that *P. acnes* [23] and porphyrins [10] are highly detectable around the surface of nose in a human face.

### Porphyrin Production in *P. acnes* Serves as a Parameter of UV Radiation

**Figure 2:**
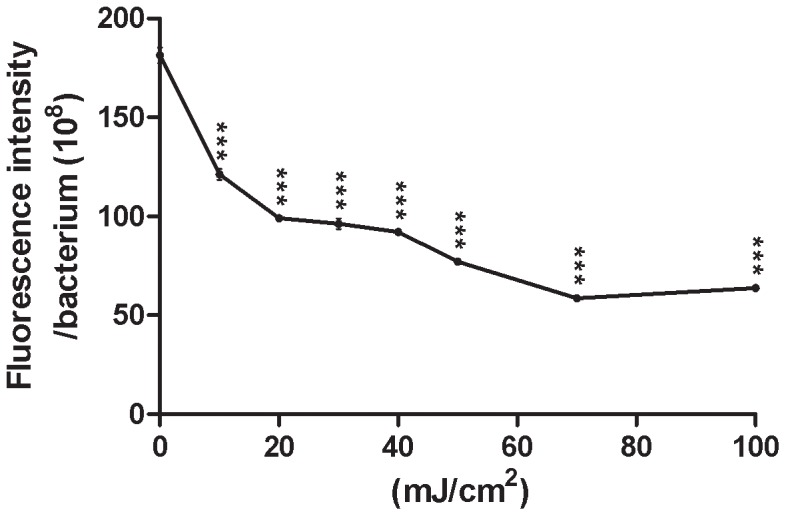
The production of porphyrins in *P. acnes* as a function of the doses of UV-B. After radiation with or without UV-B, *P. acnes* was then incubated with ALA (1 mM) for 4 h at 42°C under dark conditions. The ALA induced porphyrins were monitored using the fluorescence emission spectra via a Perkin Elmer LS50B fluorescence spectrometer. The number of bacteria was determined by reading the values of OD_600_ as described in Materials and Methods. The production of porphyrins in individual bacteria was calculated by dividing fluorescent intensities of porphyrins by the number of bacteria. ***P<0.001 was evaluated using two-tailed *t*-tests. Data are the mean ± SD of three separate experiments.

We next examine if porphyrin production in *P. acnes* can serve as a radiation parameter. *P. acnes* was exposed to UV-B at doses from 0 to 100 mJ/cm^2^. The porphyrin production of individual bacteria was monitored at 405 nm excitation and 620 nm emission by a fluorescence spectrometer and presented as a function of various doses of UV-B. As shown in Figure 2, the porphyrin production of individual *P. acnes* was reduced with increasing doses of UV-B. The amount of solar UV-B reaching the surface of the earth is 0.3 to 0.5 mW/cm^2^ at sea level, which is equivalent to a dose of 18 to 30 mJ/cm^2^ every min [2], [20]. Although the highest dose (100 mJ/cm^2^) of the UV-B used in this study theoretically can be reached 3.3–5.6 min after sunbathing at sea level, it may have actually corresponded to several hours of sunbathing when the biological effects of UV-B are taken into account [9], [24].

### UV Radiation does not Shift the Emission Peaks of Porphyrins

**Figure 3:**
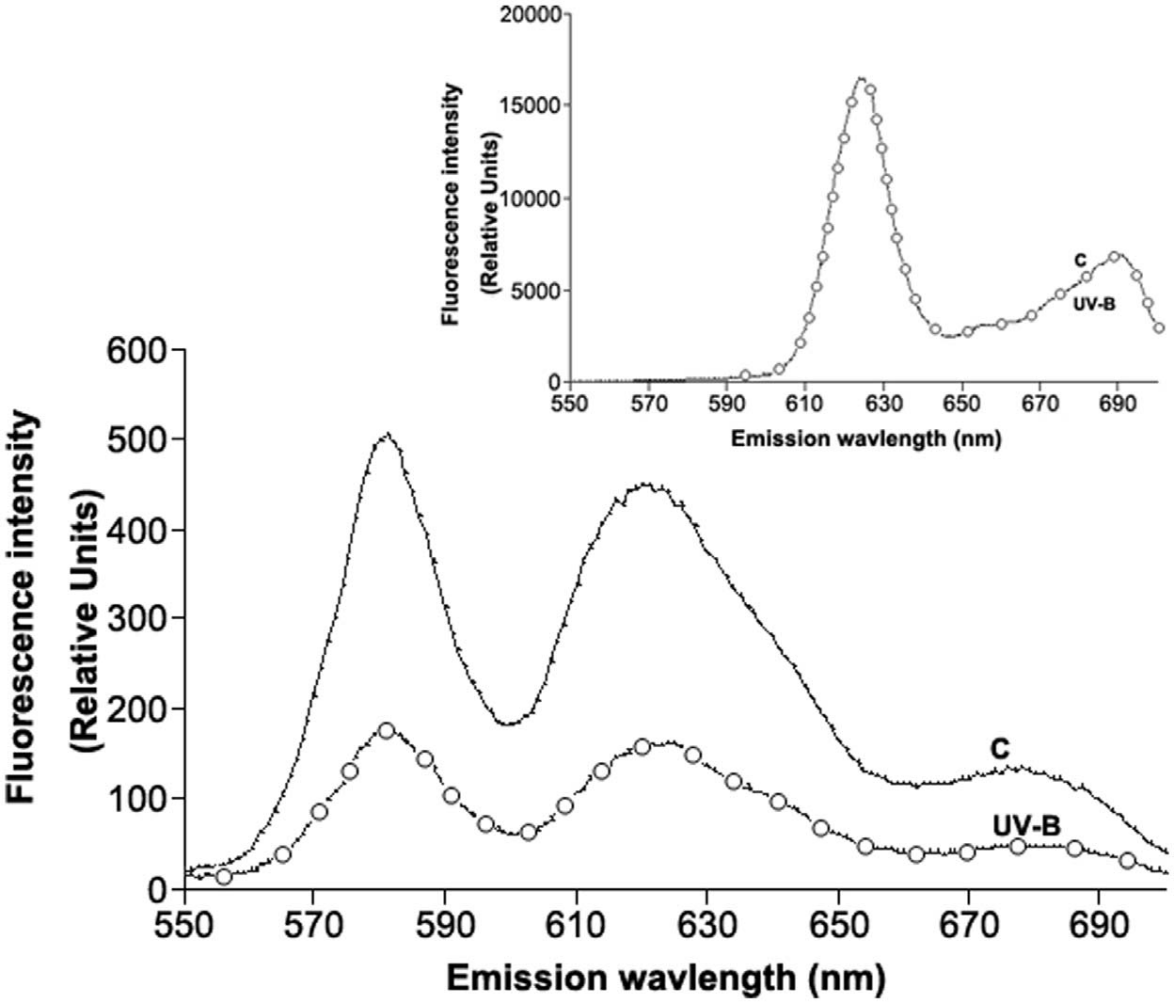
UV-B exposure did not alter the fluorescence emission spectra of porphyrins. *P. acnes* (2×10^8^ CFU) were incubated with 1 mM ALA for 4 h at 42°C and then exposed to UV-B (50 mJ/cm^2^) (UV-B). Bacteria without UV-B exposure served as a control (C). The porphyrin derivatives were detected in a spectrum between 550 and 700 nm. A spectrum of CpIII (10 µM) (Sigma, St. Louis, MO) exposed to UV-B [0 (C) or 50 mJ/cm^2^ (UV-B)] was presented in an inserted panel. Spectra are representative of three independent experiments.

The reduction of porphyrin detection in the UV-B exposed bacteria may be due to the possibility that UV-B exposure changes the chemical structures of porphyrins, leading to a shift of an emission peak. To rule out this possibility, the porphyrins produced in the ALA pre-incubated *P. acnes* were monitored in the spectrum between 550 and 700 nm (Figure 3) after exposure to UV-B at the dose of 50 mJ/cm^2^. Two major spectral peaks were found at about 580 nm and 620 nm. The 620 nm peak was ascribed to CpIII and uroporphyrin III5 [22], [25]. The peak at 580 nm has been attributed to metalloporphyrins being formed [25]. It is expected that the metalloporphyrins are formed from either free-base porphyrins by magnesium ions present in the culture medium, or by integration of zinc ions originating from the glassware. Although the fluorescence intensities of both peaks at 580 nm and 620 nm in the UV-B exposed bacteria were lower than those in bacteria without UV-B exposure, both peaks in emission spectra did not shift to other wavelengths after UV-B exposure. Similarly, exposure of CpIII to UV-B (50 mJ/cm^2^) did not alter its emission peak at 620 nm (Figure 3, inserted panel). These results suggest that UV-B exposure did not cause a significant change in the molecular structures of porphyrin derivatives.

### UV Radiation Reduces the Porphyrin Production in Human Facial Bacteria

**Figure 4:**
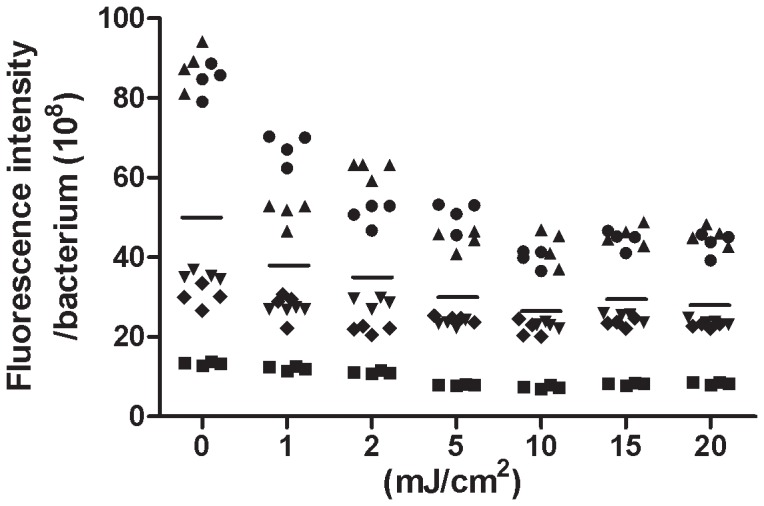
UV-B exposure decreased the production of porphyrins in bacteria isolated from human faces. Tape trips were used to isolate facial bacteria from the nose surfaces of five volunteers (▴, •, ▾, ♦, and ▪). Bacteria were exposed to UV-B ranging from 0 to 20 mJ/cm^2^ and incubated with ALA (1 mM) for 4 h at 42°C. The production of porphyrins in individual bacteria was calculated as described in Materials and Methods. The mean values [fluorescence intensity/bacterium (10^8^ CFU)] of porphyrin production in individual bacteria at a single dose of UV-B exposure were denoted ―.

*P. acnes* is a key member of the human commensals and accounts for about 50% of the total skin microbiome [9], [10]. To determine if UV-B exposure influences the production of porphyrins in *P. acnes* residing in the human facial skin, facial bacteria were isolated from five volunteers and exposed to different doses of UV-B. To examine if the bacterial response is sensitive enough to detect the effect of low UV radiation doses, human facial bacteria were irradiated UV-B ranging from 0 to 20 mJ/cm^2^. As shown in Figure 4, UV-B induced a dose-dependent reduction of porphyrin production in human facial bacteria, although the basal amounts of porphyrins in the non-irradiated individual bacterium varied from person to person.

### Concurrent Measurement of the Responses of *P. acnes* and Skin Cells to UV-B Exposure in Mice

To compare the responses of *P. acnes* and skin cells to UV-B, we inoculated *P. acnes* onto ears of Institute of Cancer Research (ICR) mice and exposed *P. acnes*-inoculated mice to UV-B. The production of porphyrin in *P. acnes* and formation of cyclobutane pyrimidine dimers (CPDs), an indication of DNA damage, in skin cells are simultaneously measured *in vivo*. 10^5^ CFU of *P. acnes* was inoculated onto mouse ear skin to recapitulate the commensal status of *P. acnes* on the surface of human skin. The outbred ICR mice were chosen because they are polymorphic at a significant number of loci and have a complex genetic history similar to a human population [26]. To minimize the effect of variations in bacterial numbers on porphyrin production, we quantified the porphyrin amounts in single colonies, not bacterial population, of *P. acnes* isolated from mouse ears. Although porphyrins may be produced by mouse cells, they will not be detected in single bacterial colonies on an agar plate, eliminating the interference effect of host porphyrins on the detection of *P. acnes* porphyrin production.

**Figure 5:**
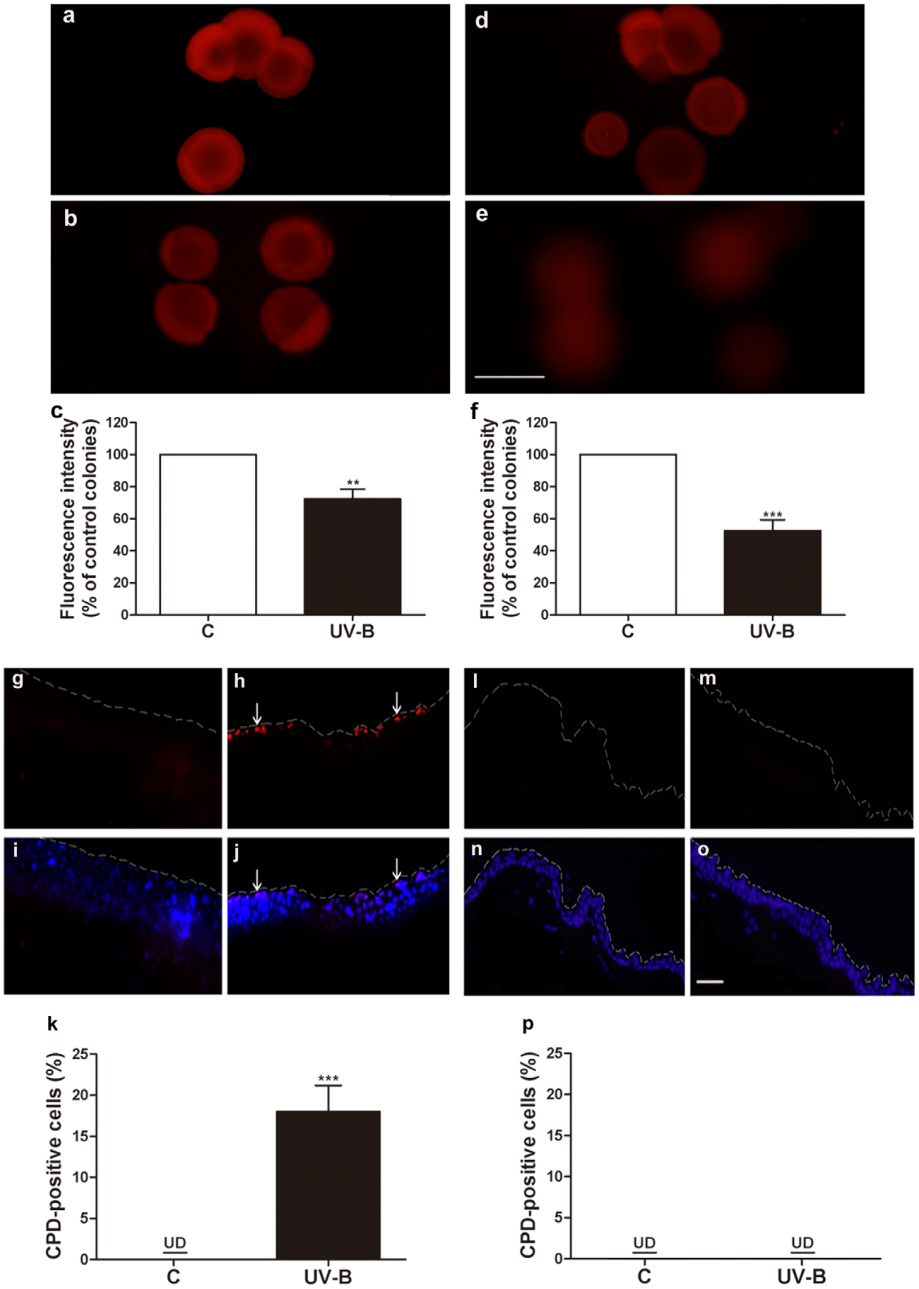
Simultaneous detection of *in vivo* responses of both *P. acnes* and skin cells to UV-B exposure. Mouse ears harboring *P. acnes* were irradiated with (50 mJ/cm^2^) and without UV-B. Ears were excised and homogenized immediately (a–c; g–k) and 24 h (d–f; l–p) after radiation. *P. acnes* in mouse homogenates was incubated with 1 mM ALA and grown on a Brucella broth agar plate. Compared with unexposed control (C) mouse ear (a, d), the intensity of porphyrins in a single colony of *P. acnes* significantly decreased after UV-B exposure (UV-B) (b, e). The decrease in the porphyrin intensity is still detectable 24 h after UV-B exposure. The intensity of porphyrin fluorescence was presented as % of that in control *P. acnes* colonies (c, f). No CPDs were detected in an epidermal layer of UV-B-unexposed-ear skin (g, i, l, n). Immunohistochemical staining showed that the CPD formation (red; arrows) detected by a monoclonal antibody to TDM-2 was considerably increased by UV-B (h, j). Merged images showed DAPI (blue) (h) and CPD (purple) (j) staining. However, UV-B-induced CPDs faded away 24 h after UV-B exposure. The percentages of CPD-positive cells in epidermal layers were displayed (k, p). Dash lines indicate the surface of ear skin. Bar (a, b, d, e) = 200 µm; (g–j; l–o) = 50 µm. **P<0.01 or ***P<0.001 was evaluated using *t*-tests. Data are the mean ± SD of three separate experiments.

As shown in Figure 5, exposure of UV-B (50 mJ/cm^2^) to *P. acnes*- inoculated mouse ears resulted in an approximately 28% decrease in porphyrin production in a single colony of *P. acnes* (Figure 5 b and c) and simultaneously induced the CPD formation in the epidermal layers of mouse skin (Figure 5 h, j and k). One day after UV-B exposure, the decrease in porphyrin production is still detectable (Figure 5 e and f), whereas CPDs disappeared from epidermal skin (Figure 5 m, o, and p). The result suggests that porphyrin reduction in *P. acnes* has a longer detection time than CPD formation in skin.

### UV Radiation Alters the Signature of Protein Oxidation in *P. acnes*

**Figure 6:**
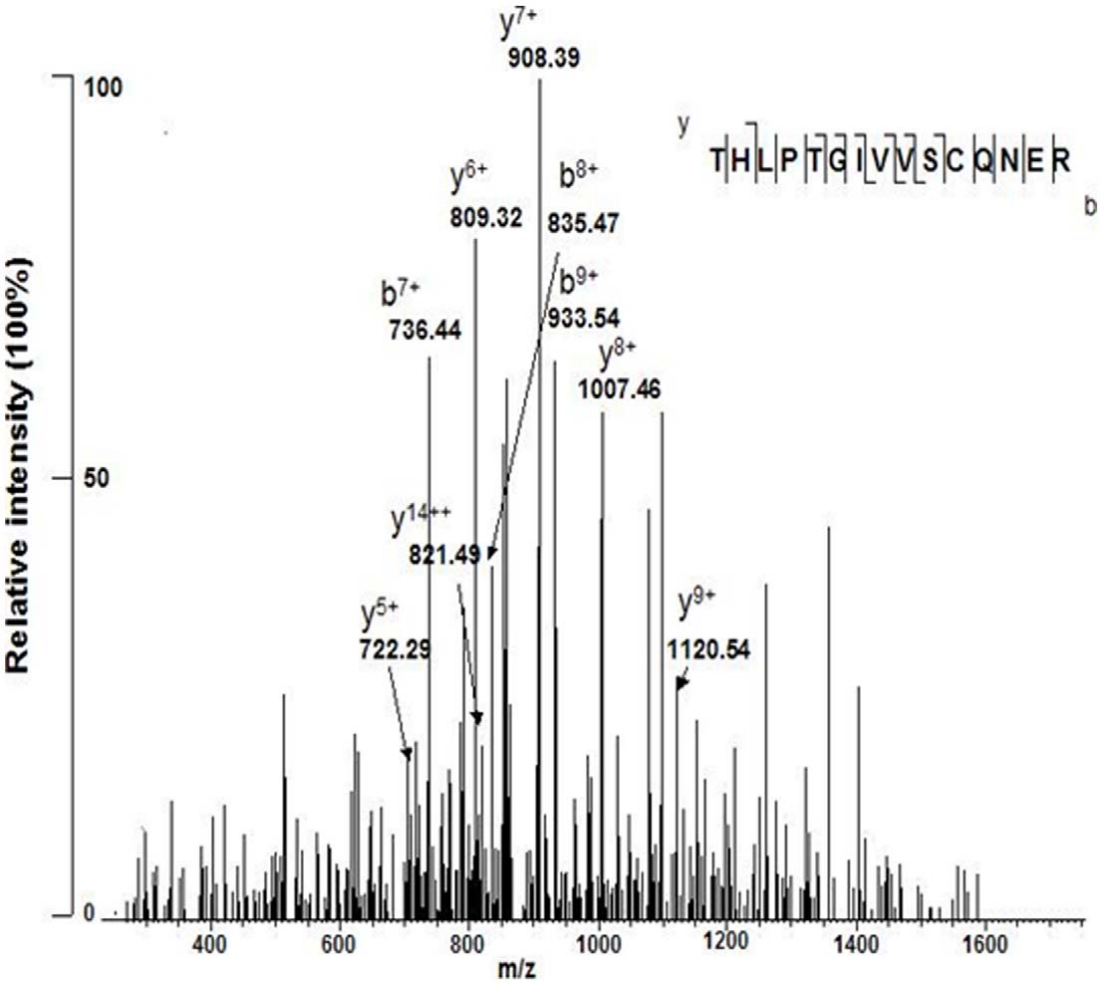
The MS/MS spectrum of an internal peptide (THLPTGIVVSCQNER) of *P. acnes* RF2. After exposure of *P. acnes* in PBS with and without UV-B at a dose of 50 mJ/cm^2^, bacterial pellets were digested with trypsin and subsequently subjected to LTQ-Orbitrap XL mass spectrometry analysis. A sequenced peptide (THLPTGIVVSCQNER) is presented and assigned as an internal peptide of *P. acnes* RF2 (Q6A808). The m/z value of each “y” and “b” ion in collision-induced dissociation (CID) spectra was indicated. Three independent experiments were performed. The oxidized THLPTGIVVSCQNER at H and C is reproducibly and exclusively present in the UV-B irradiated-*P. acnes*.

When *P. acnes* is bombarded with UV radiation, porphyrins absorb the UV and produce free radicals in return [27], [28]. In addition, it has been demonstrated that protein oxidation in bacteria can be as the primary determinant of bacterial reaction to radiation [29], [30]. Thus, we determine if UV-B radiation changes the oxidation/de-oxidation status of proteins in *P. acnes*. To establish the signature of oxidation/de-oxidation, *P. acnes* was digested with trypsin immediately after exposure to UV-B at a dose of 50 mJ/cm^2^. Trypsin-digested peptides of *P. acnes* then were subjected to reverse-phase liquid chromatography/tandem mass spectrometry (LC/MS/MS) analysis, using Michrom Paradigm high-performance liquid chromatography (HPLC) with Magic C18 column and a linear trap quadrupole (LTQ)-Orbitrap XL mass spectrometer. More than three thousand tryptic digests derived from greater than six hundred proteins in *P. acnes* exposed with and without UV-B were analyzed (data not shown). Five peptides corresponding to five *P. acnes* proteins were found oxidized after UV-B exposure (Table 1). Seven oxidized peptides derived from five *P. acnes* proteins were de-oxidized after UV-B exposure. For examples, an internal peptide (TH[153]LPTGIVVSC[176]QNER; m/z = 871.92; 263–277 amino acid residues) of a peptide chain release factor 2 (RF2) (Q6A808) with oxidation of amino acid histidine (H) and cysteine (C) was exclusively detected in UV-B irradiated *P. acnes*. The peptide (LTHLPTGIVVSCQNER; m/z = 608.65; 262–277 amino acid residues) of RF2 without oxidization was detected in *P. acnes* without radiation exposure. The MS/MS spectrum of (TH[153]LPTGIVVSC[176]QNER) was illustrated in Figure 6. The methionine (M) oxidation in three internal peptides of 60 kDa chaperonin 1 (Q9K2U4) was de-oxidized after UV-B exposure (Table 1).

**Table 1:** Mass spectrometric analysis of oxidized and de-oxidized peptides in UV-B irradiated *P. acnes*.

Oxidized peptides				
	Accession #	Peptide sequence	Amino acid #	Corresponding protein
1	Q6A808	TH[153]LPTGIVVSC[176]QNER	263–277	Peptide chain release factor 2 (RF2)
2	Q6A6L4	RPVM[147]VDPVYGSPLVSQLVSK	10–29	30S ribosomal protein S7
3	Q6A7J7	SGEQPAEAEPM[147]PDWER	234–249	30S ribosomal protein S2
4	E4HTY9	HVEIPEQVVSVNDDVM[147]VK	347–364	30S ribosomal protein S1
5	Q6A6R1	GLVLSSEEDEPVAM[147]	82–95	DNA-directed RNA polymerase subunit alpha
De-oxidized peptides				
6	Q9K2U4	RGLEAGM[147]NTLADAVK	13–27	60 KDa chaperonin 1
7	Q9K2U4	AANDEYVDM[147]VEAGIIDPAK	477–495	60 KDa chaperonin 1
8	Q9K2U4	ASISAADPTVGEIIAEAM[147]DK	147–166	60 KDa chaperonin 1
9	E4HTY9	GFLPASLVEM[147]R	149–159	30S ribosomal protein S1
10	Q6A652	FIQM[147]YGEVVEGIDAH	137–151	Pyruvate, phosphate dikinase
11	Q6A9P1	GDM[147]AVECPLEEVPLIQK	235–251	Pyruvate kinase
12	Q6A948	GLGEAM[147]VGINVADVPAPHR	255–273	Pyridoxal biosysthesis lyase pdx S

### *P. acnes* RF2 and 60 kDa Chaperonin 1

Polypeptide chain termination is the final step in protein biosynthesis, leading to peptidyl tRNA hydrolysis to release the nascent polypeptide from the ribosome. In bacteria, two codon-specific protein factors contribute to the termination reaction. Release factor 1 (RF1) catalyzes termination at UAG and UAA codons, and RF2 catalyzes termination at UGA and UAA codons [31]. The genes (*prfB*) encoding RF2 in both gram-negative and gram-positive bacteria have been cloned and characterized [32], [33], [34]. Although the biological function of *P. acnes* RF2 is unknown, UV-B-induced the oxidation of *P. acnes* RF2 may influence the protein translation in *P. acnes*. The *P. acnes* RF2 (Q6A808) shares a 45% amino acid identity with *S. epidermidis* (ATCC1228) RF2 (gi/27467454). Although the internal sequence (THLPTGIVVSCQNER) of *P. acnes* RF2 has homology with that (THHPTGIVVNNQNER) of *S. epidermidis* RF2, the cysteine amino acid is absent in the internal sequence of *S. epidermidis* RF2. Although the mechanism of de-oxidation of 60 kDa chaperonin 1 is undetermined in this study, it has been known that oxidation of chaperone plays a role in maintaining its chaperone activity [35]. De-oxidation of chaperones in *P. acnes* may be one of endogenous processes during UV damage.

### Porphyrin Reduction and Protein Oxidation/de-oxidation in Response to Gamma Radiation

*P. acnes* (ATCC6919) was exposed to gamma (^60^Co) radiation with the intention of comparing the porphyrin production and protein oxidation/de-oxidation of *P. acnes* in response to non-UV radiation. Similar to the effect of UV-B on porphyrin production (Figure 2), the porphyrin content in individual *P. acnes* was reduced with increasing doses of gamma radiation [1 to 100 gray (Gy)] (Figure S1), indicating that porphyrin reduction may be broadly inducible by other radiations. To compare protein oxidation/de-oxidation signatures of *P. acnes* in response to gamma radiation, *P. acnes* was exposed to 10 Gy of gamma radiation in phosphate buffered saline (PBS). Bacteria in PBS without radiation serve as a control. After exposure to gamma radiation, trypsinized bacteria were then analyzed by LTQ-Orbitrap XL mass spectrometry as described in Figure 6. More than three thousand tryptic digests derived from greater than six hundred proteins in *P. acnes* with and without gamma radiation exposure were analyzed. An internal peptide of a Lsr2 family protein (Q6AB31), neither RF2 nor 60 kDa chaperonin 1, was oxidized in gamma irradiated *P. acnes* (Figure S2). The result indicates that the oxidative/de-oxidative signatures can be applied to distinguish ionizing and non-ionizing radiations in *P. acnes.* Although both UV-B and gamma radiation can cause a reduction in the production of porphyrins, they may generate different signatures of protein oxidation/de-oxidation of *P. acnes*. Our future work will include establishing a UV-B-specific oxidative/de-oxidative signature by examining its changes in time- and dose-dependent manners.

## Discussion

The rationale of using the responses of *P. acnes* as radiation biomarkers includes that *1) P. acnes* resides on the human skin surface with a high density; *2)* the bacteria receive the same radiation exposure as human body; *3)* bacterial responses to radiation are less affected by internal physiological conditions of the humans; *4)* sample collection from the skin surface is readily accessible and no trained personnel required; and *5)* the response of live *P. acnes* on human faces can be monitored in a real time manner. As shown in Figure S1, UV radiation at a high dose (100 mJ/cm^2^) can completely kill *P. acnes*. However, the detected porphyrins in bacterial suspensions (Figure 1) were not those released from UV-killed bacteria because the released porphyrins were removed by re-suspension of centrifuged *P. acnes* in Reinforced Clostridium Medium (RCM) after UV-B exposure. The bacteria re-suspended in fresh RCM were grown for 24 h and then incubated with ALA for additional 4 h before detection of prophyrins. Although the enzymes that convert ALA to porphyrins may remain active in dead bacteria, a decrease in the intensity of porphyrins in a single colony of UV-irradiated *P. acnes* was detected (Figure 5), indicating that production of porphyrins was reduced in live bacteria. As shown in Figure 1, red fluorescence of porphyrins in human faces can be visualized by a Wood's lamp. It has been reported that the red fluorescence area correlated with the amount of facial sebum secretion and the skin surface pH, suggesting that the red fluorescence was affected by sebum, not just *P. acnes* [36]. Besides *P. acnes*, there are other skin resident bacteria (e.g. *Staphylococcus aureus* [37] and *Corynebacterium minutissimum* [38]) which can produce porphyrins. It has been reported that *S. epidermidis* can produce porphyrins when incubated with ALA [39], [40]. However, we are unable to detect the porphyrins in *S. epidermidis* (ATCC1228), a strain isolated from human skin. The discrepancy between studies may be because different strains of *S. epidermidis* are used, oxygen is required for porphyrin synthesis [39] or the amounts of porphyrins in *S. epidermidis* are far less than those in *P. acnes* and are below the detectable level of fluorescence microscopy. Human oral bacteria such as *Porphyromonas gingivalis* also produce porphyrins [41]. Imaging of porphyrin-producing bacteria has been used for diagnosis of various skin and oral diseases [42], demonstrating the potential clinical applications of bacterial porphyrin analysis.

The burden in humans caused by irradiation is expressed as the dose to induce minimal erythema (MED) on unirradiated skin. For untanned caucassian humans 1 MED equals 20–70 mJ/cm^2^ UV-B, depending on the skin type [43]. Since the UV-B-induced decrease of porphyrins in human facial bacteria is detectable at doses lower than 20 mJ/cm^2^, the response of *P. acnes* to UV-B may occur before significant skin injury is detected. Sun radiation may not evenly reach all *P. acnes* residing on the surface of human skins. Thus, sampling *P. acnes* from the same surface areas (e.g. nose) of human bodies may be necessary when the measurement of UV-B response in *P. acnes* is applied clinically. The number of *P. acnes* residing on the surface of human skins varies from individual to individual. It has been reported that the density of *P. acnes* paralleled age- and gender-related differences in sebaceous gland activity [44], [45], although our result indicated that host gender might not a significant factor for porphyrin production in bacteria (Figure 4). To circumvent the problem related to variation in numbers of bacteria, we have quantified the amounts of porphyrins in individual *P. acnes* (Figure 2), individual bacteria isolated from human faces (Figure 4) and a single bacterial colony isolated from *P. acnes*-inoculated mouse ear (Figure 5). Although the presence of DNA damage has been used as a UV radiation biomarker, the sensitivity of CPDs is cell type-dependent and induction of CPDs is transient and reversible due to a rapid repair response to DNA damage in cells [46]. As shown in Figure 5, the duration of porphyrin reduction is longer than that of CPD formation after UV-B exposure. Thus, analysis of porphyrin reduction in *P. acnes* may provide a stable biomarker for radiations. Each individual’s skin may react differently when exposed to UV-B. Six skin phototypes (Type I to VI) are defined by the wide range of different sensitivities to sunlight [47]. The ranges of MED for type I (burns easily) and V (never burns) are 15–30 and 90–150 mJ/cm^2^ UV-B, respectively [47]. It has been reported that higher CPD yields in the skin of normal human with higher UV-B sensitivity [48], [49]. Although the reduction of porphyrin production in a UV dose-dependent manner was detectable in five facial bacteria isolated from humans (Figure 4), it is worth studying the correlation of porphyrin reduction with CPD formation and skin sensitivity to UV-B.

Oxidation can occur under normal physiological conditions [50] and during sample preparation [51]. However, oxidized peptides caused by non-specific factors during normal conditions and sample preparation have been subtracted after comparing the mass spectra of oxidized proteins in *P. acnes* with and without radiation exposure. The 60 kDa chaperonin 1 (Q9K2U4) of *P. acnes* shares a 61% amino acid identity with *S. epidermidis* (ATCC1228) chaperonin GroEL 60 kDa (gi/27468547). Methionines of internal peptides (AANDEYVDMVEAGIIDPAK and ASISAADPTVGEIIAEAMDK) of *P. acnes* 60 kDa chaperonin 1 are also present in the corresponding peptides (AATNEWVNMLEEGIVDPT and GAISAADEEIGRYISEAMDK) of *S. epidermidis* chaperonin GroEL 60 kDa. However, methionine of the internal peptide (RGLEAGMNTLADAVK) of *P. acnes* 60 kDa chaperonin 1 is absent in the corresponding peptide (QAMLRGVDKLANAVK) of *S. epidermidis* chaperonin GroEL 60 kDa. Thus, it is worthy to investigate if the cysteine oxidation of THLPTGIVVSCQNER and methionine oxidation of RGLEAGMNTLADAVK are UV-B-specific responses exclusively found in *P. acnes*.

Noninvasive sampling bacteria on the uppermost layers of skin will simplify the process of environmental and accidental radiation detection based on the response of *P. acnes*. Thus, the reaction of *P. acnes* to UV-B, which affects the surface layers of skin, is investigated in this study. Both UV-B and 400 nm lights exist in the solar spectrum. The 400 nm light can penetrate deep into skin and hair follicles and has been used in phototherapy to kill *P. acnes* [52]. In addition, the absorption spectrum of porphyrins exhibits a maximum at 400 nm. Proteins can undergo a wide variety of oxidative post-translational modifications (oxPTM); while reversible modifications are thought to be relevant in physiological processes, non-reversible oxPTM may contribute to pathological situations and disease [53]. Thus, it is critical to investigate if the responses of *P. acnes* to UV are permanent and irreversible after radiation exposure. Since people often use antioxidant-containing skin products, it is worth determining if antioxidants decrease the extent of oxidation/de-oxidation of *P. acnes* proteins. In addition, it is possible that *P. acnes* in human skins has become UV resistant [54] during evolution. However, the production of porphyrins in human facial bacteria containing *P. acnes* was decreased with the increasing doses of UV-B (Figure 4), suggesting that the human facial bacteria are responsive to UV radiation.

Since *P. acnes* resides on the surface of human faces, real-time detection of oxidized/de-oxidized-*P. acnes* peptides in humans without collecting any samples will be useful for rapidly screening the potential victims of radiation exposure and prompt treatment decisions in clinics. A recent study demonstrated the noninvasive imaging of oxidized low-density lipoprotein in atherosclerotic plaques with tagged oxidation-specific antibodies [55]. Potential application of this noninvasive image includes early diagnosis of lipid-rich atherosclerotic lesions. Similar to this concept, the tagged oxidized/de-oxidized-*P. acnes*-specific antibodies can be applied onto the skin (face or fingertip) of humans to diagnose the radiation exposure. To our knowledge, we are the first group to propose the idea of using molecular responses of human skin microbiome to external environments as biomarkers for human diseases. To support this idea, we measured the porphyrin production and established an oxidative/de-oxidative proteome of *P. acnes* in response to radiation with an aim to predict the future risk of cancer. Although both UV-B and gamma radiation reduced the production of porphyrins (Figures 2 and S1), they may induce different signatures of protein oxidation/de-oxidation of *P. acnes* (Figures 6 and S2). Skin cancer may result from long term effects of UV exposure while injury caused by gamma radiation can be an acute radiation response. Our future work will include establishing a UV-B-specific oxidative/de-oxidative signature by examining its changes in time (acute versus long term)- and dose-dependent manners. Both external factors, such as use of antioxidant containing skin products, and intrinsic factors, such as age, may lead to inter-individual variations in the oxidative/de-oxidative signatures. Thus, future work will also include investigating the effects of these factors on protein oxidation/de-oxidation of *P. acnes.* For clinical applications, standardization of *P. acnes* sampling procedures for analytical detection is required to reduce the intra- and inter-individual variability of the skin microbiome [56].

Significance in this study includes *1)* providing a new application and understanding of human skin microbiome in health and disease; *2)* monitoring the responses of skin commensal microbes to radiation as predictive markers, as response markers, or as surrogate endpoints for cancer imaging and therapy. These markers would provide guidance on the quantities of drugs or radiation [e.g. radiation therapy and computed tomography (CT) scan using gamma radiation] required and the optimal time of application; and *3)* facilitating the development of skin microbiome-based biodosimetry for detection of the risk of radiations in a battlefield, space, terror attacks and nuclear accidents.

## Materials and Methods

### Ethics Statement

Experiments of using mice were performed at University of California, San Diego (UCSD). The UCSD ethics committee specifically approved this study under an approved Institutional Animal Care and Use Committee (IACUC) protocol (no. S10058). The written consents from all participants were obtained before conducting tape strip sampling. The Institutional Review Board (IRB) at UCSD approved the consent procedure and sampling using tape strips under an approved protocol (no. 100473).

### Bacterial Culture

*P. acnes* (ATCC 6919) (Manassas, VA) was grown on a Brucella broth agar (BD, Sparks, MD) under anaerobic conditions using a Gas-Pak (BD, Sparks, MD) at 37°C as described in our previous publication [57]. Single colonies were inoculated in RCM (Oxford, Hampshire, England) and grown at 37°C until reaching the logarithmic growth phase with a value of optical density (OD_600_) at 1.0. *S. epidermidis* (ATCC 12228) was grown on a Tryptic soy broth (TSB) (Sigma, St. Louis, MO) agar overnight at 37°C. The bacteria from single colonies were cultured in TSB overnight at 37°C until reaching around OD_600_ = 1.0. The bacteria were centrifuged at 5,000×g for 10 min, washed with PBS, and suspended to appropriate amount of PBS for the experiments.

### Detection of Porphyrin-producing *P. acnes*

To visualize porphyrin-producing bacteria, *P. acnes* (2×10^8^ CFU) or *S. epidermidis* (2×10^8^ CFU) was incubated with ALA (Sigma, St. Louis, MO) for 4 h and observed under the Bx51 research microscope (Olympus, Melville, NY, USA.) and X-Cite 120 fluorescence illumination systems (EXFO, Quebec, Canada). For imaging of auto-fluorescent *P. acnes* in human facial skin, a Wood's lamp (SkinMate. Tulsa, OK) with an UV light source was used to locate the *P. acnes* on the skins of volunteers according to the methods as previously described [36].

### Quantification of Porphyrins in Humans

A D-Squame Standard Sampling Discs adhesive tape strip (CuDerm Corporation, Dallas, TX, USA) with a diameter of 2.0 cm was applied to the exposed site on noses, foreheads, and arms of five volunteers (5 males aged 25–35 years) and removed using clean forceps after a 10 sec adhesion time. The adhesive tape strip was removed slowly with constant force at am approximately 45° angle. Four successive tape strips (five in total) were carefully applied to the same site immediately after the previous tape strip was removed and the new strip was be also retained on the skin for 10 sec. The tape strip was rolled with the adhesive side facing out, placed into a 2 ml cryovial containing 1 mM ALA for 24 h at 42°C under dark conditions, and then sonicated for 2 min. ALA was dissolved in dimethyl sulfoxide (DMSO) to a concentration of 500 mM as a stock solution. The ALA induced porphyrins were monitored using the fluorescence emission spectra via a Perkin Elmer LS50B fluorescence spectrometer. The excitation was set to 405 nm and the fluorescence emission was monitored at 620 nm. CpIII dissolving in a buffer solution (PIPES 20 mM, KCl 100 mM, pH 7.2) was used as internal standard for quantification of porphyrins. A calibration curve was obtained and found linear from 4 to 500 nM.

### UV-B Exposure in Mice

*P. acnes* (ATCC 6919) (10^5^ CFU in 20 µl PBS) bacteria were epicutaneouly applied onto the dorsal ear skins of ICR mice for 10 min. Mouse ears harboring *P. acnes* then were irradiated with (50 mJ/cm^2^) or without UV-B. To determine the effect of UV-B exposure on the production of porphyrins in *P. acnes*, mouse ears harboring *P. acnes* were excised, homogenized, incubated with 1 mM ALA overnight, and then spread on a Brucella broth agar plate immediately and 24 h after UV-B exposure. Porphyrins in *P. acnes* colonies were observed under the Bx51 research microscope (Olympus, Melville, NY, USA.) and X-Cite 120 fluorescence illumination systems (EXFO, Quebec, Canada). To quantify the intensity of porphyrins in a single colony of *P. acnes*, the total intensities of porphyrins in a field with approximately 50 colonies were calculated by ImageJ software and divided by the number of bacterial colonies. Three fields from three separate experiments were randomly selected for calculation. For detection of CPDs, mouse ears harboring *P. acnes* were excised right after UV-B exposure, embedded in Tissue-Tek O.C.T. (Sakura Finetek, Netherlands), and subjected to immunohistochemical staining based on the protocol as described previously [58] using primary mouse monoclonal antibodies against CPD (TDM-2). The diamidino-2-phenylindole (DAPI) was used as a nuclear counterstain. Mouse ears harboring *P. acnes* without UV-B exposure served as controls. Specimens were observed using a Bx51 research microscope (Olympus, Melville, NY, USA.) in conjunction with X-Cite 120 fluorescence illumination systems (EXFO, Quebec, Canada). The percentage of CDP-positive cells in an epidermal layer of mouse ear skin was calculated from approximately 100 cells in a microscopic field. Three fields from three independent experiments were randomly chosen for calculation.

### Change in the Porphyrin Production in UV-B-exposed Bacteria

A UV-B lamp (BLE-IT158 Spectronics Corporation, Westbury, New York, USA) with emission between 280 and 320 nm, a spectrum peak at 312 nm, and fluence rate at 4 W/m^2^ was used. The *P. acnes* (ATCC 6919) (2×10^8^ CFU in PBS) or bacteria (2×10^8^ CFU in PBS) isolated from tape-tripped human facial skins of five volunteers (3 males aged 22–47 years and 2 females aged 26–38) were spread on 100 mm culture dishes (BD Biodcirnvrdg, NC, USA) and positioned 15 cm under a UV-B lamp during exposure at doses from 0 to 100 mJ/cm^2^. For gamma radiation, the *P. acnes* (ATCC 6919) (2×10^8^ CFU in PBS) was irradiated with gamma radiation (^60^Co, Siemens Theratron Elite 80). After exposure with or without radiation, bacteria in PBS were spun down and re-suspended in RCM for further growth. After 24 h growth, the number of bacteria was determined by reading OD at 600 nm as measured by a spectrophotometer (Eppendof Bio photometer, Hamburg, Germany). Subsequently, bacteria were washed twice after centrifugation (2,500 g, 5 min) and re-suspended in a buffer solution containing piperazine-N,N′-bis(2-ethanesulfonic acid) (PIPES) 20 mM, KCl 100 mM, pH 7.2. Bacteria were then incubated with ALA (1 mM) for 4 h at 42°C under dark conditions. Porphyrins were monitored using the fluorescence emission spectra as described above. The production of porphyrins in individual bacteria was calculated by dividing fluorescent intensities of porphyrins by the number of bacteria. The porphyrin derivatives were detected in the spectrum between 550 and 700 nm.

### LTQ-Orbitrap XL Mass Spectrometry Analysis

After exposure of *P. acnes* in PBS with (50 mJ/cm^2^ UV-B or 10 Gy gamma radiation) and without radiation, pellets of bacteria were re-suspended in 100 µl of 50 mM ammonium bicarbonate. Tris(2-carboxyethyl)phosphine (TCEP) was added to 10 mM and incubated for 20 min. Then iodoacetamide was added and incubated for 35 min. Proteins were digested with trypsin (10 ng/µl; Promega, Madison, WI, USA) was overnight at 37°C and subsequently subjected to reverse-phase LC/MS/MS analysis, using Michrom Paradigm HPLC with Magic C18 column and a LTQ-Orbitrap XL mass spectrometer (Thermo Scientific, Portsmouth, NH, USA) as described in details in our previous publication [59].

### Statistical Analysis

Data are presented as mean ± standard deviation (SD). The Student *t*-test was used to assess the significance of independent experiments. The criterion *P*<0.05 was considered statistically significant.

## Supporting Information

Figure S1 **The production of porphyrins in** ***P. acnes*** **is decreased with increasing the doses of gamma radiation.** After radiation with or without (0, 1, 2, 5, 10, 100 Gy) gamma radiation (^60^Co), *P. acnes* was then incubated with ALA (1 mM) for 4 h. The ALA induced porphyrins were monitored using the fluorescence emission spectra via a Perkin Elmer LS50B fluorescence spectrometer as described in Figure 2. The number of bacteria was determined by reading the values of OD_600_. The production of porphyrins in individual bacteria was calculated by dividing fluorescent intensities of porphyrins by the number of bacteria. ***P<0.001 was evaluated using two-tailed *t*-tests. Data are the mean ± SD of three separate experiments.
(TIF)

Figure S2 **Mass spectrometric sequencing of a peptide in** ***P. acnes*** **Lrs2.** Protein oxidation/de-oxidation of *P. acnes* irradiated with and without 10 Gy gamma radiation was identified by LTQ-Orbitrap XL mass spectrometry as described in Materials and Methods. A sequenced peptide (DALSLWVDHAR) is presented and assigned as an internal peptide of a *P. acnes* Lsr2 family protein (Q6AB31). The m/z value of each “y” and “b” ion in CID spectra was indicated. Three independent experiments (n = 3) were performed. The oxidized DALSLWVDHAR at W and H is reproducibly and exclusively present in the *P. acnes* irradiated with gamma radiation.
(TIF)

Figure S3 **The viability of** ***P. acnes*** **after UV-B exposure.** *P. acnes* bacteria were exposed to UV-B at the doses of 20, 40, 50 and 100 mJ/cm^2^. Bacteria without UV-B exposure (0 mJ/cm^2^) served as a control. After exposure, the CFUs of *P. acnes* (1∶10–100,000 dilution) were visualized (a) and quantified (b) on Brucella broth agar plates. **P<0.001 was evaluated using student’s *t*-test. Data are the mean ± SD of three independent experiments.
(TIF)

Figure S4 **Validation of** ***P. acnes*** **in the tape-stripped samples.** DNA extracted from tape-stripped samples of three volunteers (Lanes 4–6) was amplified by PCR using primers for the 16S rRNA gene of *P. acnes*. Negative controls included the PCR reactions using pure water (Lane 1) and DNA of *S. epidermidis* (ATC12228) (Lane 2). The DNA of *P. acnes* (ATCC6919) was used as a positive control in the PCR reaction (Lane 3). The 600-bp PCR product (arrow) of 16S rRNA gene in a 1.0% agarose gel was indicated. A 1 kb DNA ladder (Lane 7) (Invitrogen, CA, USA) was used as a nucleic acid marker.
(TIF)

Supporting Information S1 (DOC)
