# Supplementary figures and images for: The Response of Human Skin Commensal Bacteria as a Reflection of UV Radiation: UV-B Decreases Porphyrin Production
Source: PLoS One. 2012 Oct 25;7(10):e47798. doi: 10.1371/journal.pone.0047798 (PMC3485044; doi:10.1371/journal.pone.0047798)

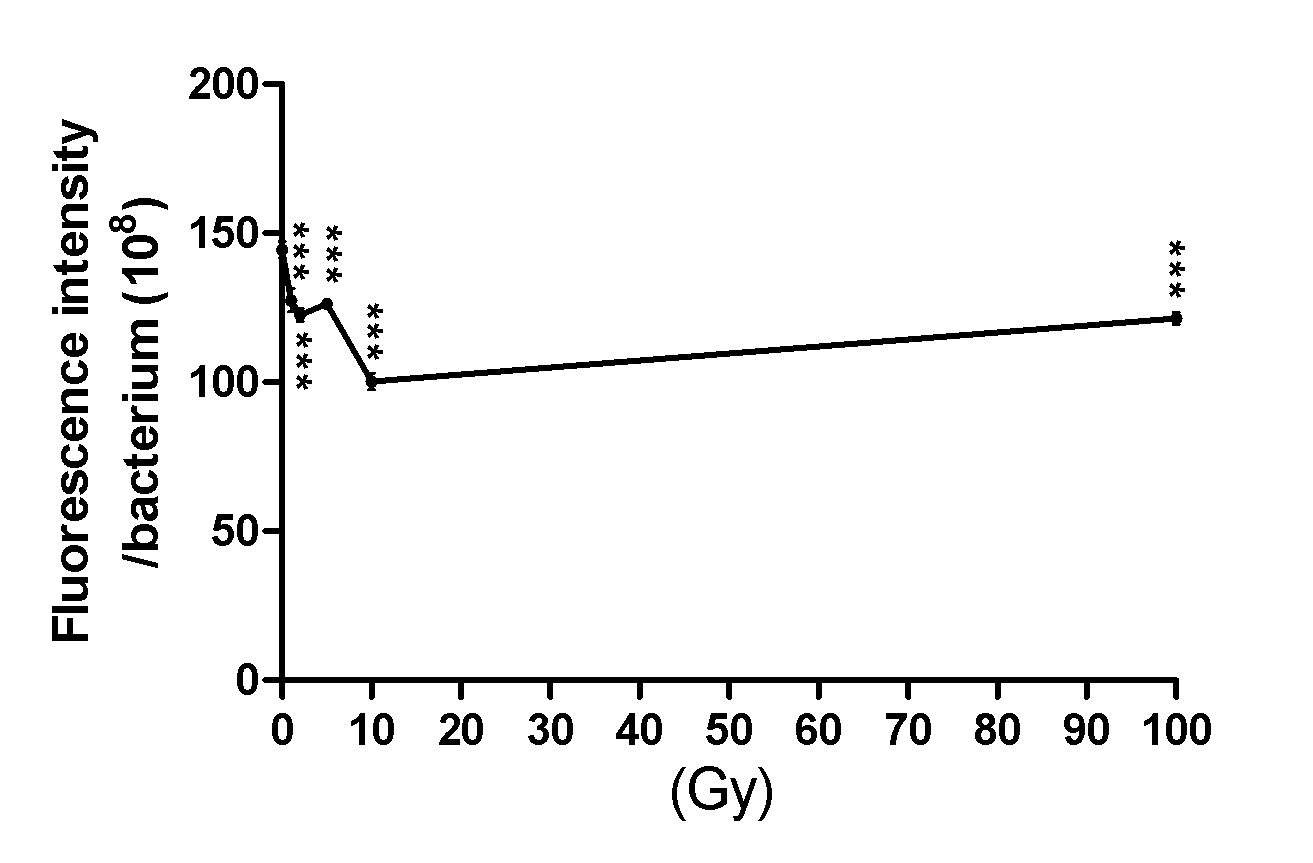

Supplement: Figure S1 — The production of porphyrins in P. acnes is decreased with increasing the doses of gamma radiation. After radiation with or without (0, 1, 2, 5, 10, 100 Gy) gamma radiation (60Co), P. acnes was then incubated with ALA (1 mM) for 4 h. The ALA induced porphyrins were monitored using the fluorescence emission spectra via a Perkin Elmer LS50B fluorescence spectrometer as described in Figure 2. The number of bacteria was determined by reading the values of OD600. The production of porphyrins in individual bacteria was calculated by dividing fluorescent intensities of porphyrins by the number of bacteria. ***P<0.001 was evaluated using two-tailed t-tests. Data are the mean ± SD of three separate experiments. (TIF) [file pone.0047798.s001.tif]

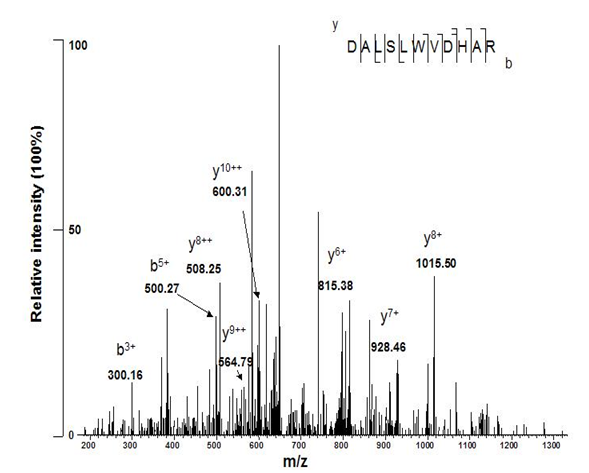

Supplement: Figure S2 — Mass spectrometric sequencing of a peptide in P. acnes Lrs2. Protein oxidation/de-oxidation of P. acnes irradiated with and without 10 Gy gamma radiation was identified by LTQ-Orbitrap XL mass spectrometry as described in Materials and Methods. A sequenced peptide (DALSLWVDHAR) is presented and assigned as an internal peptide of a P. acnes Lsr2 family protein (Q6AB31). The m/z value of each “y” and “b” ion in CID spectra was indicated. Three independent experiments (n = 3) were performed. The oxidized DALSLWVDHAR at W and H is reproducibly and exclusively present in the P. acnes irradiated with gamma radiation. (TIF) [file pone.0047798.s002.tif]

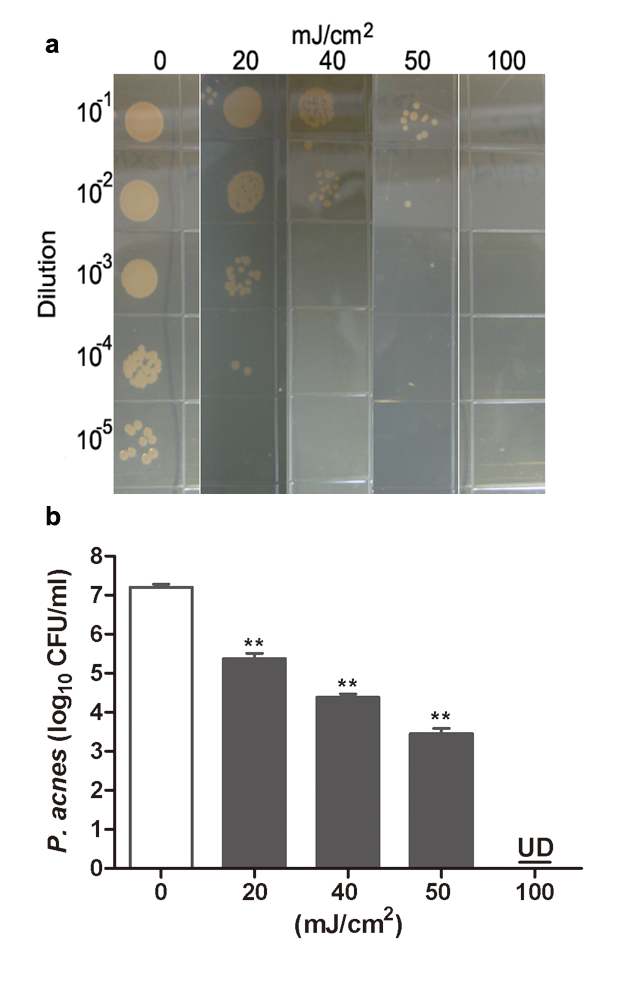

Supplement: Figure S3 — The viability of P. acnes after UV-B exposure. P. acnes bacteria were exposed to UV-B at the doses of 20, 40, 50 and 100 mJ/cm2. Bacteria without UV-B exposure (0 mJ/cm2) served as a control. After exposure, the CFUs of P. acnes (1∶10–100,000 dilution) were visualized (a) and quantified (b) on Brucella broth agar plates. **P<0.001 was evaluated using student’s t-test. Data are the mean ± SD of three independent experiments. (TIF) [file pone.0047798.s003.tif]

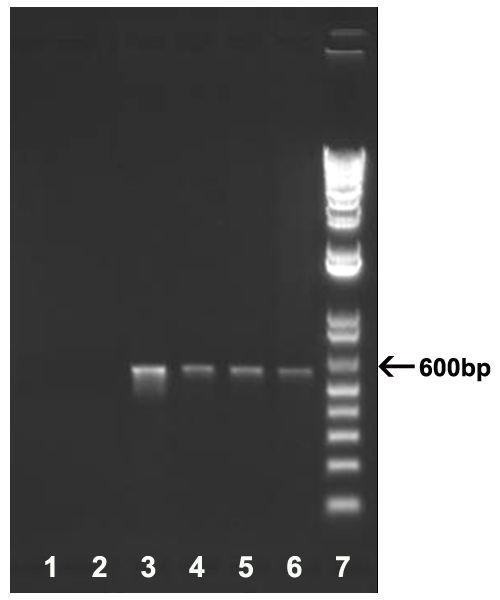

Supplement: Figure S4 — Validation of P. acnes in the tape-stripped samples. DNA extracted from tape-stripped samples of three volunteers (Lanes 4–6) was amplified by PCR using primers for the 16S rRNA gene of P. acnes. Negative controls included the PCR reactions using pure water (Lane 1) and DNA of S. epidermidis (ATC12228) (Lane 2). The DNA of P. acnes (ATCC6919) was used as a positive control in the PCR reaction (Lane 3). The 600-bp PCR product (arrow) of 16S rRNA gene in a 1.0% agarose gel was indicated. A 1 kb DNA ladder (Lane 7) (Invitrogen, CA, USA) was used as a nucleic acid marker. (TIF) [file pone.0047798.s004.tif]
