# Supplementary material for: The Response of Human Skin Commensal Bacteria as a Reflection of UV Radiation: UV-B Decreases Porphyrin Production
Source: PLoS One. 2012 Oct 25;7(10):e47798. doi: 10.1371/journal.pone.0047798 (PMC3485044; doi:10.1371/journal.pone.0047798)
Supplement: Supporting Information S1 — (DOC) [file pone.0047798.s005.doc]

**SUPPLEMENTAL INFORMATION**

Gamma radiation is the common product of radioactive atoms and can be emitted during nuclear accidents. Consensus guidance based on a threshold whole-body or significant partial body radiation exposure suggests starting antibiotics and cytokine therapy at exposure dose of 2 Gy [S1]. A significant partial body or whole-body dose >10 Gy is considered lethal. While a radiation dose has a well-characterized effect on human tissues, it is difficult to retroactively determine the amount of individual exposure after an uncontrolled and massive radiation event. The current gold-standard is to measure chromosome damage in circulating lymphocytes [S2]. The drawback of this method includes that such chromosomal abnormality is unstable and short lived because the cells carry such chromosome defects will be eliminated from the circulation [S3]. Other disadvantages include that once chromosome abnormality is detected, radiation induced-gene mutation may have been started. Several biomarkers of gamma radiation have been identified, but the detection of these biomarkers in tissues, such as blood, may require invasive sampling techniques. Collection of biological fluids (such as urine) presents a noninvasive way for identification of radiation biomarkers, but fluid secretion may be a late response of humans to radiation [S4]. We envision that the bacteria residing in human skin serve as an endogenous and always-on radiation sensor. Due to its high density in human skin, *P. acnes* was selected as model bacteria to investigate the response of skin commensal bacteria to UV-B (Figures 1, 2, 5, and 6) and gamma radiation (Figures S1 and S2). Results in Figures 2 and 5 demonstrated that UV-B exposure significantly decreased the porphyrin production of *P. acnes*. Here we found that gamma radiation, like UV-B, induced a dose-dependent decrease in the porphyrin production of *P. acnes* (Figure S1). An oxidized peptide (DALSLWVDHAR) derived from a Lsr2 family protein was detectable exclusively in *P. acnes* irradiated with 10 Gy gamma radiation (Figure S2).

Theoretically, reduction in the number of bacterial counts may be able to reflect the amount of radiation exposure. As shown in Figure S3, UV-B exposure suppressed the growth of *P. acnes* in a dose-dependent manner. Exposure of UV-B to *P. acnes* (1.6 x 10^7^ ± 3.1 x 10^6^ CFU) at the dose of 20 mJ/cm^2^ resulted in a considerable decrease (2.3 x 10^5^ ± 8.8 x 10^4^ CFU) in the number of bacterial counts. UV-B exposure at the dose of 100 mJ/cm^2^ completely suppressed the growth of *P. acnes*. Although UV-B exposure reduced the bacterial growth, the density of *P. acnes* residing on the surface of human skins is different from one person to another, making it difficult to use the bacterial reduction as a parameter for radiation exposure. We thus examine if porphyrin production in *P. acnes* can serve as a radiation parameter in this study.

To ensure that *P. acnes* is one of microbes in human bacterial isolates, the polymerase chain reaction (PCR) for detection of 16S rRNA genes of *P. acnes* was performed (Figure S4). The 16S rRNA genes of *P. acnes* were detected in the human bacterial isolates, *P. acnes* (a positive control), but not *S. epidermidis* (a negative control), indicating that *P. acnes* is present in microbes isolated from tape-tripped human facial skins.

**SUPPLEMENTAL MATERIALS AND METHODS**

**The effect of UV-B on the growth of *P. acnes***

*P. acnes* bacteria (2 x 10^8^ CFU in PBS) spread on culture dishes (100 mm diameter) (BD Falcon, NJ, USA) were exposed to UV-B lamps at doses of 0, 20, 40, 50 and 100 (mJ/cm^2^). After exposure, the CFUs of *P. acnes* were determined by spotting 5 μl of the dilution (1:10-100,000) on a Brucella broth agar plate as previously described [56].

**Detection of *P. acnes* by PCR**

The DNA in tape-stripped samples was extracted by following the instructions in the Wizard® Genomic DNA purification Kit (Wizard®; Promega, Madison, WI, USA). PCR were performed with a DNAEngine (BioRad®) Peltier Thermal cycler. Specific primers were used for the detection and amplification of 131 base pairs of the 16S rRNA gene of *P. acnes*. The primer sequences were: a forward primer: 5'-GGGTTGTAAACCGCTTTCGCC-3' and a reverse primer: 5'-GGCACACCCATCTCTGAGCAC-3'. A cycle protocol was employed for the following conditions: initial 94ºC denature for 2 min; 30 cycles of 94ºC denature for 30 sec, 60ºC annealing for 1 min and 72ºC extension for 1 min.; 72ºC extension for 7 min; 4ºC hold. PCR products were confirmed by gel electrophoresis on 1.0% agarose.

**REFERENCES**

S1. Mitchel RE, Jackson JS, Morrison DP, Carlisle SM (2003) Low doses of radiation increase the latency of spontaneous lymphomas and spinal osteosarcomas in cancer-prone, radiation-sensitive Trp53 heterozygous mice. Radiat Res 159: 320-327.

S2. Bender MA, Awa AA, Brooks AL, Evans HJ, Groer PG, et al. (1988) Current status of cytogenetic procedures to detect and quantify previous exposures to radiation. Mutat Res 196: 103-59.

S3. Elisova TV (2008) Stable and unstable chromosome aberrations in humans and other mammals in relation to the problems of biological dosimetry. Radiats Biol Radioecol 48: 14-27.

S4. Sperati A, Abeni DD, Tagesson C, Forastiere F, Miceli M, et al. (1999) Exposure to indoor background radiation and urinary concentrations of 8-hydroxydeoxyguanosine, a marker of oxidative DNA damage. Environ Health Perspect 107: 213-215.
